# Monitoring H-cluster assembly using a semi-synthetic HydF protein†
†Electronic supplementary information (ESI) available. See DOI: 10.1039/c8dt04294b


**DOI:** 10.1039/c8dt04294b

**Published:** 2019-01-03

**Authors:** Brigitta Németh, Charlène Esmieu, Holly J. Redman, Gustav Berggren

**Affiliations:** a Molecular Biomimetics , Department of Chemistry – Ångström Laboratory , Uppsala University , 75120 Uppsala , Sweden . Email: gustav.berggren@kemi.uu.se

## Abstract

The [FeFe] hydrogenase enzyme interconverts protons and molecular hydrogen with remarkable efficiency. The reaction is catalysed by a unique metallo-cofactor denoted as the H-cluster containing an organometallic dinuclear Fe component, the [2Fe] subsite. The HydF protein delivers a precursor of the [2Fe] subsite to the apo-[FeFe] hydrogenase, thus completing the H-cluster and activating the enzyme. Herein we generate a semi-synthetic form of HydF by loading it with a synthetic low valent dinuclear Fe complex. We show that this semi-synthetic protein is practically indistinguishable from the native protein, and utilize this form of HydF to explore the mechanism of H-cluster assembly. More specifically, we show that transfer of the precatalyst from HydF to the hydrogenase enzyme results in the release of CO, underscoring that the pre-catalyst is a four CO species when bound to HydF. Moreover, we propose that an electron transfer reaction occurs during H-cluster assembly, resulting in an oxidation of the [2Fe] subsite with concomitant reduction of the [4Fe4S] cluster present on the HydF protein.

## Abstract 2

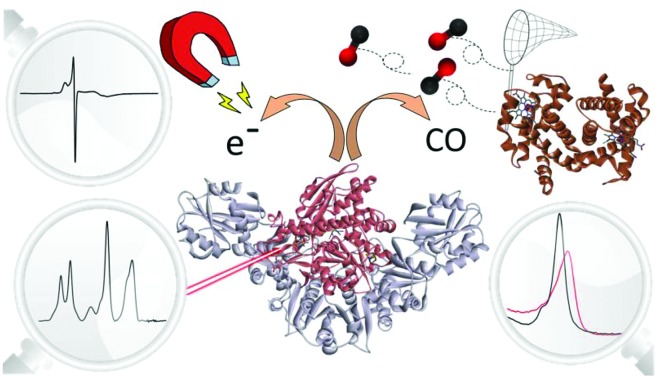
An artificial enzyme, incorporating an organometallic iron complex, is used to probe the activation mechanism of [FeFe] hydrogenase.

## Introduction

**Fig. 1:**
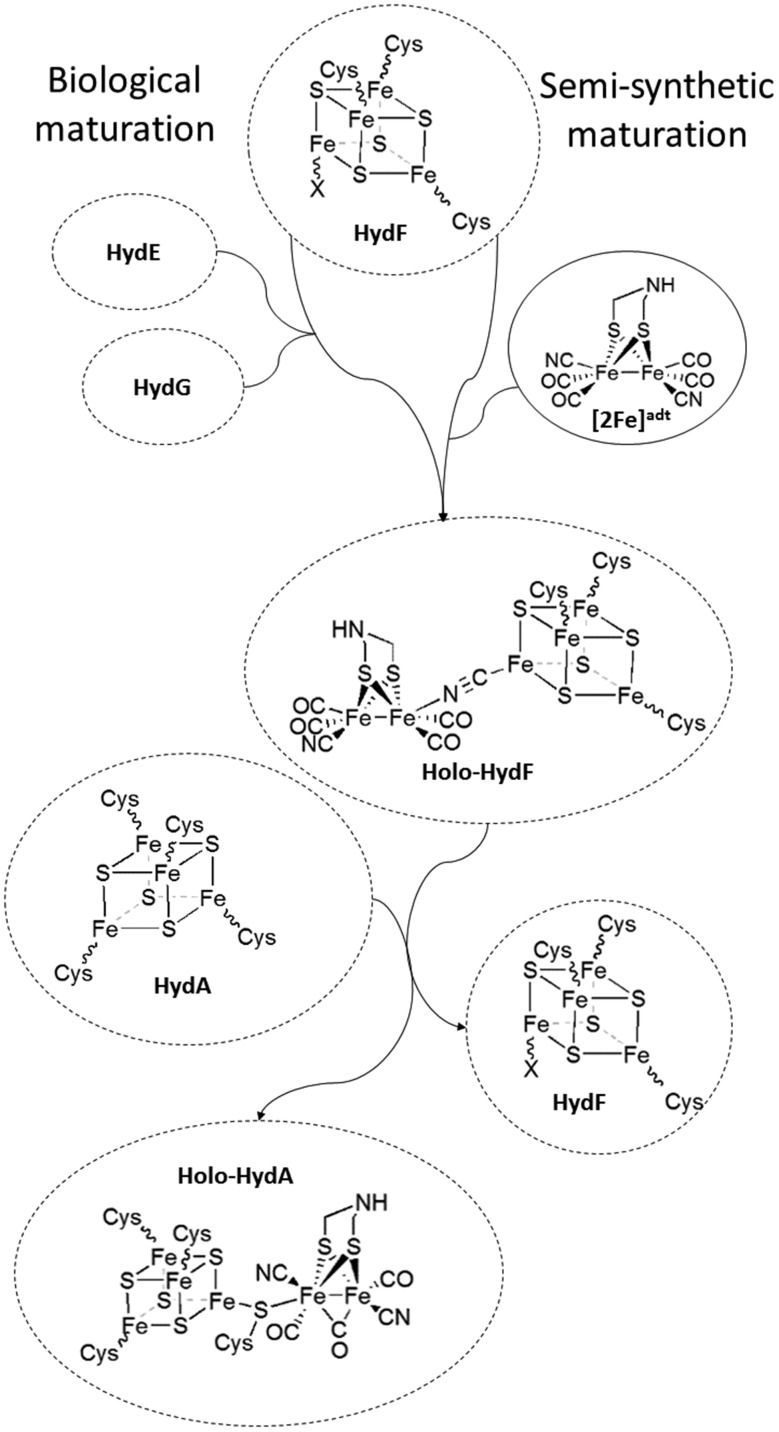
Schematic representation of the assembly of holo-HydF ([2Fe]^adt^-HydF) and formation of the H-cluster. During the biological process, a pre-catalyst is assembled on HydF through the activities of HydE and HydG. In the semi-synthetic route, holo-HydF is generated *via* the incorporation of synthetic mimics of the [2Fe] subsite.

The [FeFe] hydrogenases (HydA) are ancient metalloenzymes that catalyze the reversible interconversion between protons and molecular hydrogen, utilizing a hexanuclear iron cofactor called the “H-cluster”.^1^,^2^ The H-cluster is composed of a canonical [4Fe4S] cluster ([4Fe4S]_H_) coupled to a low valent diiron complex, the [2Fe] subsite, *via* a single bridging cysteine derived thiol ligand. The Fe ions of the [2Fe] subsite are bridged by an aza-dithiolate ligand (–SCH_2_NHCH_2_S–, adt), and further coordinated by carbonyl and cyanide ligands.^3^–^6^ The assembly of the H-cluster is a two-step process, where the biosynthesis of the [2Fe] subsite and its insertion into apo-hydrogenase^7^ requires at least three hydrogenase specific auxiliary proteins or maturation enzymes.^8^–^12^ Two of these enzymes (HydG and HydE) belong to the radical *S*-adenosyl-l-methionine (SAM) enzyme family. HydG has been shown to catalyze the formation of the diatomic CO and CN ligands from tyrosine in a SAM dependent reaction.^13^–^18^ The substrate and the product of HydE is still not fully elucidated.^19^,^20^ The third protein, HydF, contains a [4Fe4S] cluster and displays GTPase activity.^21^–^24^ The role of HydF is to harbor and deliver the assembled [2Fe] subsite precursor to apo-HydA, thus completing the H-cluster (Fig. 1). The exact nature of this pre-catalyst, and the mechanism of H-cluster assembly remains unclear. Still, it is well established that purified forms of HydF from *Clostridium acetobutylicum*, isolated from cells co-expressing HydE and HydG (CaHydF^EG^), are capable of activating apo-HydA.^22^,^25^ Moreover, FTIR spectra recorded on CaHydF^EG^ show CO and CN^–^ signals reminiscent of those observed for the hydrogenase enzyme.^25^–^27^ These observations show that HydF expressed in this way contains all the components necessary to activate the enzyme, *i.e.* a complete precursor of [2Fe] subsite, with no further modifications from other maturation enzymes required.

Recent studies have shown that the synthetic [2Fe] subsite mimic [Fe_2_(adt)(CO)_4_(CN)_2_]^2–^ ([2Fe]^adt^), can be introduced into HydF ([2Fe]^adt^-HydF). Spectroscopic and biochemical characterization of this artificially matured HydF has shown that the [2Fe]^adt^ complex retains its (i,i) oxidation state and binds to the protein *via* the [4Fe4S] cluster, thus providing a model for “holo-HydF” (Fig. 1).^5^,^28^ Critically, this semi-synthetic form of HydF can mimic the reactivity of native HydF, transfer its synthetic cargo to apo-HydA, and generate a fully active form of HydA indistinguishable from the native [FeFe] hydrogenase enzyme. This approach provides a convenient tool for the preparation of large quantities of homogenous and active HydF for structural and mechanistic studies. However, the biological relevance of these semi-synthetic forms of HydF remains to be verified.

Prior work on semi-synthetic HydF has employed proteins from thermophilic organisms, *i.e. Thermotoga maritima* (TmHydF) and *Thermosipho melanesiensis* (TmeHydF). Herein we report the preparation of the semi-synthetic form of HydF from *C. acetobutylicum* ([2Fe]^adt^-CaHydF) allowing a direct comparison to biological references (*i.e.* CaHydF^EG^). Moreover, we show using CO-release assays and a combination of EPR and FTIR spectroscopy that the pre-catalyst on HydF features four carbonyl ligands and is resistant to potent chemical oxidants. Finally, we demonstrate that the transfer of the pre-catalyst from [2Fe]^adt^-CaHydF to apo-HydA results in the oxidation of the [2Fe] complex to generate the H-cluster in a mixture of oxidized states, the so-called H_ox_ and H_ox_-CO states.

## Experimental

### General

All chemicals were purchased from Sigma-Aldrich or VWR and used as received unless otherwise stated. Protein purity was analyzed by 12% SDS-PAGE minigels in a SE250 Mighty Small II unit (Hoefer) system. The proteins were stained with Page Blue protein staining solution (Thermo Fisher Scientific) according to the supplier instructions. Protein concentrations were determined with Comassie Plus (Bradford) assays (Thermo Fischer Scientific), using bovine serum albumin as a standard. All anaerobic work was performed in an MBRAUN glovebox ([O_2_] < 10 ppm). The expression vector encoding the *csdA* gene was kindly provided by Prof. Marc Fontecave (Collège de France, Paris/CEA, Grenoble). The His-tagged cysteine desulfurase enzyme, CsdA, was purified following a literature procedure.^29^ Bovine hemoglobin was purchased from Sigma-Aldrich. The EPR and FTIR spectra shown are representative signals from at least two individual experiments. UV/vis spectra recorded in the glovebox were recorded using an Avantes AvaSpec-ULS2048-USB2-UA-50 spectrometer equipped with fibre optics and all other UV/Vis spectra recorded using an Agilent Cary 50 UV/Vis spectrometer.

The (Et_4_N)_2_[Fe_2_(adt)(CO)_4_(CN)_2_] ([2Fe]^adt^) complex was synthesized in accordance to literature protocols with minor modifications, and verified by FTIR spectroscopy.^30^,^31^ The complex was dissolved in anaerobic buffer (100 mM Tris-HCl pH 8.0, 300 mM KCl, 25 mM MgCl_2_) at 2.4 μg μL^–1^ (3.7 mM) concentration and used directly.

### Protein expression and purification

The expression and isolation of *Chlamydomonas reinhardtii* HydA1 (CrHydA1) and CaHydF, was performed according to literature procedures with minor modifications.^26^,^32^–^34^ All steps related to expression and isolation were performed under aerobic conditions. Constructs encoding *C. acetobutylicum hydF* and *C. reinhardtii hydA1* genes were transformed into chemically competent *E. coli* Rosetta (II) (Stratagene) cells. Fresh plates were streaked and single colonies were chosen for small scale overnight culture growth in media supplemented with 100 μg mL^–1^ ampicillin and 33 μg mL^–1^ chloramphenicol. The following morning the seed cultures were used to inoculate 6 L of media which comprised 10 g L^–1^ tryptone, 5 g L^–1^ yeast extract, 5 g L^–1^ KCl, 5 g L^–1^ glucose, 25 mM potassium phosphate buffer pH 7.4, 100 μg mL^–1^ ampicillin and 33 μg mL^–1^ chloramphenicol. Cell cultures were grown at 37 °C and 230 rpm shaking until the optical density at 600 nm (OD_600_) reached 0.5–0.6. The cultures were then induced with IPTG (0.5 mM final concentration), and shaken at 150 rpm for 16 h at 16 °C. Cells were harvested by centrifugation, and the resulting cell pellets were immediately flash frozen in liquid N_2_. Composite cell pellet mass was recorded, and cells were stored at –80 °C until further use.

#### Cell lysis

Cell pellets were thawed and resuspended in a lysis buffer containing 100 mM Tris-HCl pH 8.0, 300 mM KCl, 5% glycerol and 25 mM MgCl_2_. The cell lysis mixture was supplemented with 0.03 mg mL^–1^ DNase and 0.03 mg mL^–1^ RNase. The volume of the lysis mixture was adjusted to the optical density and the volume of the culture (5 ml lysis buffer/OD/L culture). This mixture was frozen in liquid nitrogen and thawed at room temperature in a water bath 3 times. The suspension was then discontinuously sonicated for 5 minutes. The cell debris and the membrane fraction were separated from the soluble fraction *via* ultracentrifugation (55 000 rpm 70 min 4 °C). The resulting supernatant was flash frozen in liquid nitrogen and kept at –80 °C.

#### Protein purification

The supernatant was filtered and loaded onto StrepTrap (GE Healthcare) columns previously equilibrated with Tris-HCl buffer (100 mM Tris-HCl pH 8.0, 300 mM KCl, 5% glycerol and 25 mM MgCl_2_). The columns were washed with the same buffer until the UV baseline reached zero. The elution was performed with 2.5 mM desthiobiotin supplemented equilibration buffer. Selected fractions were pooled together and concentrated using 30 kDa amicon filters and flash frozen in liquid nitrogen.

#### Demetalization

Residual metal ions were removed from the purified proteins under strict anaerobic conditions. In a standard experiment 0.5–0.8 mM of CrHydA1 or CaHydF was incubated at 4 °C in the presence of 20 molar equivalents of sodium dithionite (NaDT, 10–16 mM) and 10 molar equivalents (5–8 mM) of EDTA for 2 hours in the case of CaHydF and 3 hours for CrHydA1.

#### Size exclusion chromatography

The EDTA treated proteins were centrifuged and loaded on a previously equilibrated (100 mM Tris-HCl pH 8.0, 300 mM KCl, 5% glycerol and 25 mM MgCl_2_) Superdex200 size exclusion chromatographic column (GE Healthcare). The same buffer was used for elution at a flowrate 0.8 ml min^–1^. The elution fractions were collected and concentrated using 30 kDa centricons. The proteins were flash frozen in liquid nitrogen and stored at –80 °C until further use.

### *In vitro* reconstitution of [4Fe4S] clusters

The *in vitro* reconstitution of the [4Fe4S] clusters in CrHydA1 and CaHydF was performed as previously described.^23^,^32^ In a standard reaction HydA1 or HydF (150 μM) was incubated with l-cysteine (900 μM, 6 molar eq.), Fe(NH_4_)_2_(SO_4_)_2_ (Mohr's salt) (900 μM, 6 molar eq.), dithiothreitol (DTT) (1.5 mM, 10 molar eq.) and CsdA (3 μM, 0.02 molar eq.) in a Tris-HCl buffer (100 mM Tris-HCl pH 8.0, 300 mM KCl, 25 mM MgCl_2_). When the absorbance at ≈410 nm reached a plateau (approx. 2 h) the reaction mixture was filtered through a NAP25 desalting column (GE Healthcare). The reconstituted proteins were aliquoted into PCR tubes and transferred into serum vials. The proteins in the serum vials were flash frozen in liquid nitrogen outside of the glovebox and stored at –80 °C until further use. The presence of the [4Fe4S] cluster was verified by UV/Vis and EPR spectroscopy. The iron and sulfur content was quantified using the methods reported by Fish and Beinert, respectively, with minor modifications.^35^,^36^

### Preparation of [2Fe]^adt^-CaHydF

In a standard reaction HydF (200 μM) was treated with [2Fe]^adt^ (2.4 mM, 12 molar eq.) at room temperature, under dimmed light conditions for 1 hour. The reaction was arrested by filtering the solution through a NAP25 desalting column equilibrated with 25 mM Tris-HCl pH 8.0, 50 mM KCl and 25 mM MgCl_2_, providing a clear separation between unreacted [2Fe]^adt^ and [2Fe]^adt^-HydF. The [2Fe]^adt^-HydF containing elution fractions were pooled together, aliquoted into PCR tubes, and flash frozen analogously to the reconstituted proteins.

### H_2_ evolution assays

The hydrogen evolution assays were performed as previously reported with minor modifications.^5^ 0.04 μM [4Fe4S]-CrHydA1 was incubated at 37 °C for 60 minutes with various concentrations of [2Fe]^adt^-CaHydF (0.04–1.2 μM) in 400 μl 100 mM K-phosphate buffer pH 6.8 complemented with 2 mM Na-DT and 1 v/v% Triton X-100. After 60 minutes 1.6 ml of a reduced methyl viologen (10 mM methyl viologen, 1 v/v% Triton X-100, 100 mM Na-DT) solution was added to the protein mixture. Hydrogen gas was quantified after 15 minutes of incubation using a gas chromatograph (GC; PerkinElmer LLC, MA, USA) equipped with a thermal conductivity detector (TCD) and a stainless-steel column packed with Molecular Sieve (60/80 mesh). A calibration curve was established by injecting known amounts of hydrogen. The operational temperatures of the injection port, the oven and the detector were 100 °C, 80 °C and 100 °C, respectively. Argon was used as the carrier gas at a flow rate of 35 mL min^–1^.

### EPR sample preparation and measurement

EPR samples (generally at 200 μM protein concentration) were prepared under strict anaerobic conditions. The proteins were reduced with 10–20 fold molar equivalents (2–4 mM) of NaDT or oxidized with 10–20 fold molar equivalents (2–4 mM) of thionine acetate for 20–40 minutes. The reduction was followed by UV/Vis, monitoring the disappearance of the [4Fe4S]^2+^ absorbance around 410 nm. The samples were transferred into quartz EPR tubes, capped with rubber septa before they were removed from the glovebox and immediately flash frozen. The EPR samples were stored in liquid nitrogen.

The low temperature CW EPR measurements were carried out with a Bruker Elexys 500 X-band spectrometer using an ER049X SuperX microwave bridge in a Bruker SHQ0601 resonator equipped with an Oxford Instruments continuous flow cryostat and using an ITC 503 temperature controller (Oxford Instruments). Low temperature measurements were carried out with liquid helium as coolant. The spectrometer was controlled by the Xepr software package (Bruker). Spectra were recorded with a 10 G modulation amplitude and a 100 kHz modulation frequency. Spectra were averaged over either four or eight scans.

### FTIR sample preparation and measurement

For the FTIR measurements, a Demountable Liquid FTIR Cell (Pyke technologies) composed by calcium fluoride windows and a 25 μm Teflon spacer was used. Silicon grease was applied onto the spacer before the cell assembly, to prevent leakage and to minimize the sample volume.

**Fig. 2:**
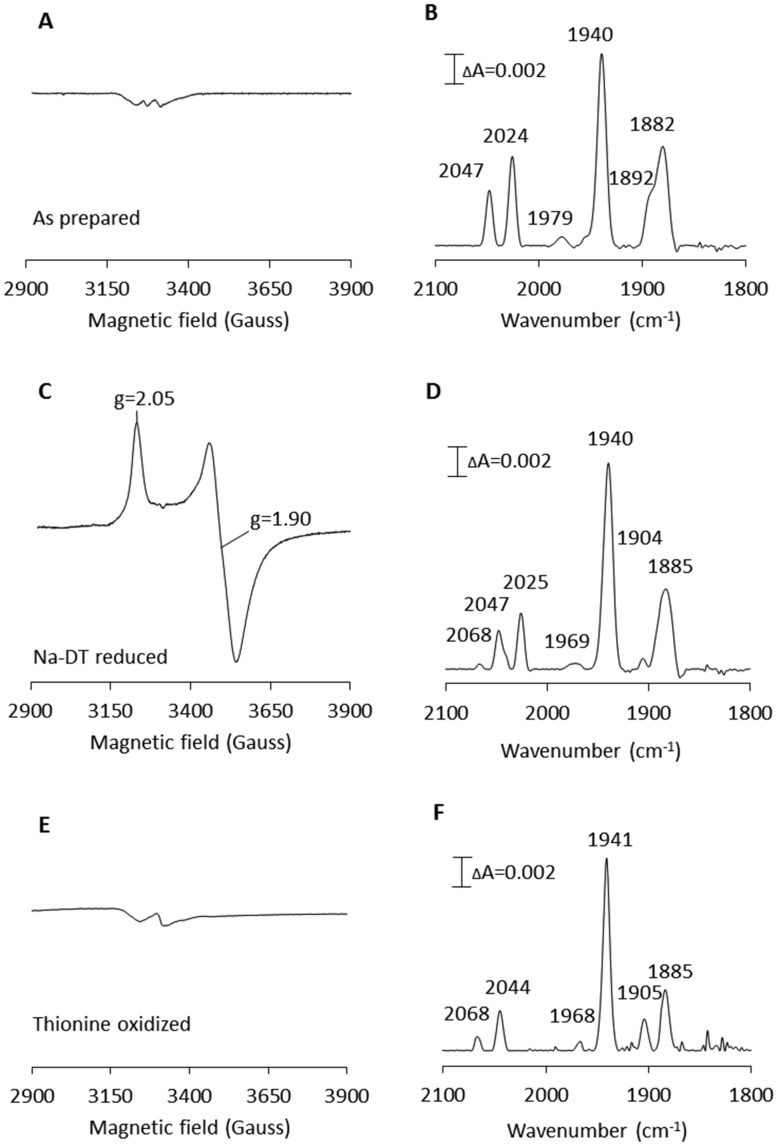
X-band EPR and FTIR spectroscopic characterization of artificially matured CaHydF, [2Fe]^adt^-CaHydF. (A) EPR spectrum recorded on as-prepared [2Fe]^adt^-CaHydF (0.2 mM); (B) FTIR spectrum recorded on as-prepared [2Fe]^adt^-CaHydF (2.5 mM); (C) EPR spectrum recorded on NaDT reduced [2Fe]^adt^-CaHydF (0.2 mM, 4 mM NaDT); (D) FTIR spectrum recorded on NaDT reduced [2Fe]^adt^-CaHydF (2.5 mM, 50 mM NaDT); (E) EPR spectrum recorded on thionine oxidized [2Fe]^adt^-CaHydF (0.25 mM, 2.5 mM thioinine); (F) FTIR spectrum recorded on thionine oxidized [2Fe]^adt^-CaHydF (2.5 mM, 25 mM thioinine). All samples were prepared in 25 mM Tris-HCl pH 8.0 50 mM KCl. EPR spectra were recorded at a microwave power of 1 mW, at 10 K, microwave frequency: 9.28 GHz.

FTIR spectra were collected with a Bruker IFS 66v/S FT-IR spectrophotometer equipped with a Bruker MCT (mercury–cadmium–telluride) detector. The interferograms were accumulated in the double-sided, forward–backward mode with 750 scans. All measurements were performed with a resolution of 2 cm^–1^. The baseline treatment was carried out using the rubber-band concave baseline treatment in Opus software (Fig. 2 and 4), and manual polynomial baseline subtraction using Origin (Fig. S1†) in order to see the effect of the baseline treatment on the FTIR spectra.

**Fig. 3:**
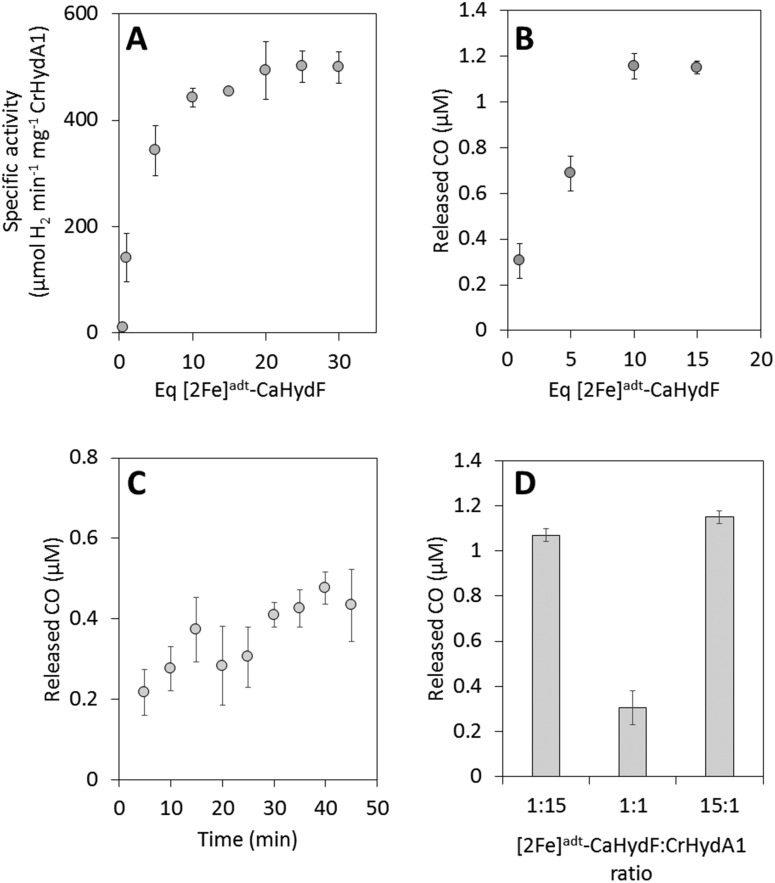
The activation of apo-CrHydA1 using [2Fe]^adt^-CaHydF probed by enzymatic assays and CO release. (A) H_2_ production assay, CrHydA1 titration curve: 0.04 μM apo-CrHydA1 (1 equivalent) was incubated with 0.04–1.2 μM of [2Fe]^adt^-CaHydF in 100 mM K-phosphate pH 6.8 buffer at 37 °C for 60 minutes. After 60 minutes, hydrogenase activity was determined *via* a methyl viologen assay. (B) CO release, CrHydA1 titration curve: 2 μM CrHydA1 (1 equivalent) was incubated with 2–30 μM of [2Fe]^adt^-CaHydF for 25 minutes. (C) Time dependence of CO release: 2 μM apo-CrHydA (1 equivalent) was incubated with 2 μM [2Fe]^adt^-CaHydF (1 equivalent) for 5–45 min. (D) CO release as a function of the relative ratio between apo-CrHydA1 and [2Fe]^adt^-CaHydF – 2 μM [2Fe]^adt^-CaHydF to 30 μM apo-CrHydA1 (1 : 15), 2 μM [2Fe]^adt^-CaHydF to 2 μM apo-CrHydA1 (1 : 1) and 30 μM [2Fe]^adt^-CaHydF to 2 μM CrHydA1. All CO release experiments were performed at room temperature in 100 mM Tris-HCl pH 7.0 300 mM KCl.

**Fig. 4:**
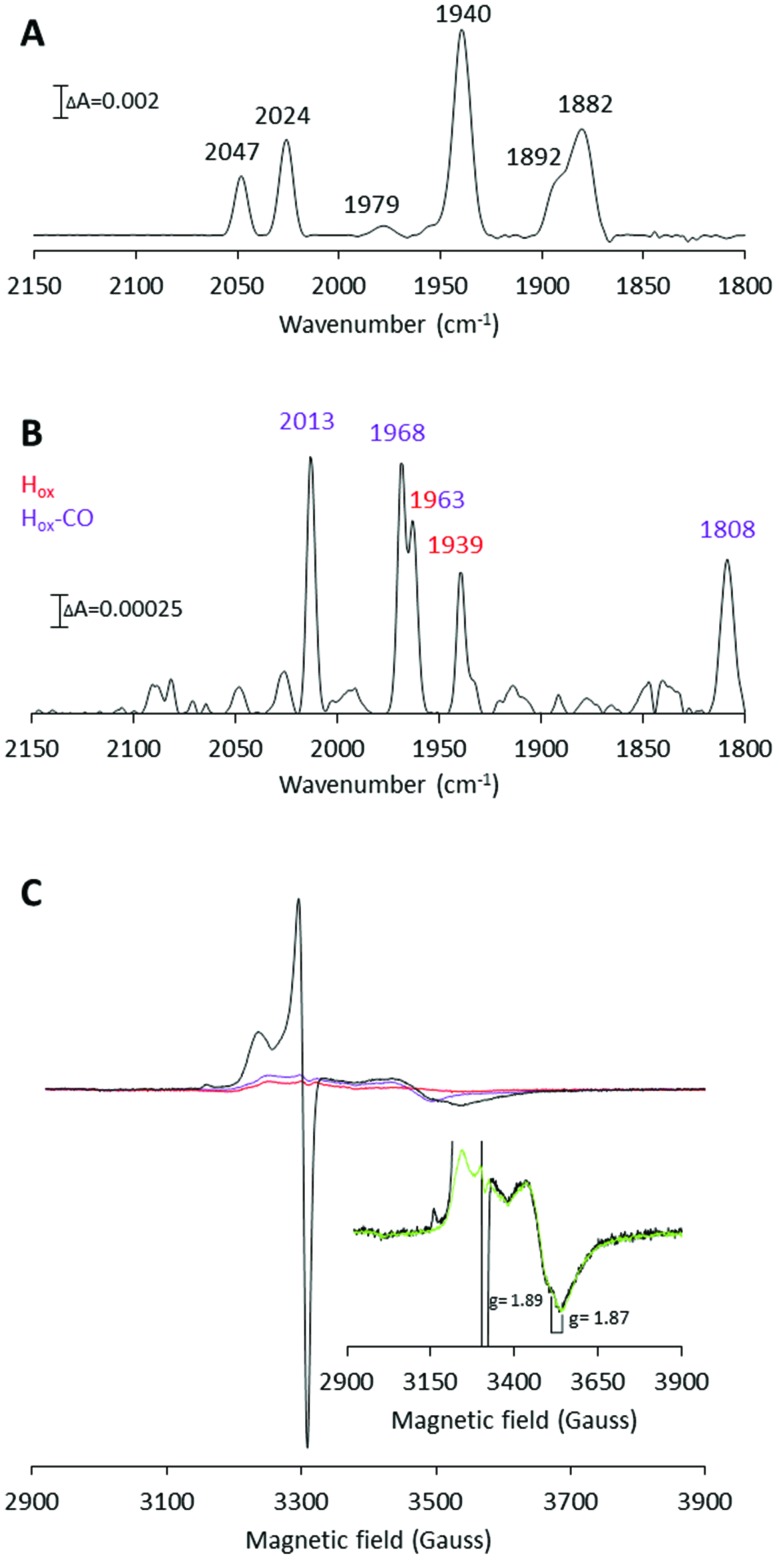
H-Cluster assembly monitored by FTIR and X-band EPR spectroscopy. (A) FTIR spectrum recorded on 2.5 mM [2Fe]^adt^-CaHydF (reproduced from Fig. 2, panel B). (B) FTIR spectrum recorded on a mixture of apo-CrHydA1 (1.5 mM) and [2Fe]^adt^-CaHydF (300 μM) after 60 min incubation in 25 mM Tris-HCl pH 7.0, 50 mM KCl. Color coding: H_ox_, red; H_ox_-CO, purple. (C) X-band EPR spectra. Black line: apo-CrHydA1 (200 μM) incubated with [2Fe]^adt^-CaHydF (30 μM) for 60 min; Red line: [2Fe]^adt^-CaHydF (30 μM); Purple line: apo-CrHydA1 (200 μM). (Inset): Black line: apo-CrHydA1 (200 μM) treated with [2Fe]^adt^-CaHydF (30 μM); Green line: a linear combination of [4Fe4S]^+^-CaHydF (16 μM) and apo-CrHydA1 (200 μM). All EPR samples were prepared in 100 mM Tris-HCl pH 7.0 300 mM KCl, and recorded at a microwave power of 1 mW, at 10 K, microwave frequency: 9.28 GHz.

### Hemoglobin assay

Bovine hemoglobin (200 μM) was reduced to deoxyhemoglobin (Hb) under strictly anaerobic conditions with 2 mM NaDT, in 100 mM Tris-HCl pH 7.0. The UV/vis spectra were recorded at room temperature in sealed micro UV-cuvettes to ensure anaerobic conditions. The CaHydF–CrHydA1 mixtures were prepared under strict anaerobic conditions in the glovebox and incubated at room temperature for varying time intervals. The hemoglobin solution was added to the CaHydF–CrHydA1 mixtures at the end of the incubation period from sealed anaerobic vials (final Hb concentration 4 μM), using gastight Hamilton syringes. After each injection, mixing was ensured by gently shaking the cuvette. Each sample had an individual baseline that was recorded right before hemoglobin addition. The formation of carboxyhemoglobin (COHb) was quantified based on the extinction coefficients of Hb (*ε*_419_ = 79 812 M cm^–1^; *ε*_430_ = 97 875 M cm^–1^) and COHb (*ε*_419_ = 138 750 M cm^–1^; *ε*_430_ = 35 625 M cm^–1^), obtained from calibration curves recorded under strict anaerobic conditions (Fig. S2†). These experimentally obtained extinction coefficients were in good agreement with previously published extinction coefficients.^16^

## Results and discussion

### Protein purification and reconstitution

The metal-free forms of both CrHydA1 and CaHydF were obtained *via* recombinant expression in *E. coli* and purified in good yields following affinity and size-exclusion chromatography under aerobic conditions (approx. 30 and 8 mg protein per L cell culture, respectively). The FeS clusters were reconstituted *via* anaerobic incubation of the proteins with a slight excess of Mohr's salt (Fe(NH_4_)_2_(SO_4_)_2_), l-cysteine and DTT in the presence of catalytic amounts of cysteine desulfurase (CsdA), as previously described for *e.g.* CrHydA1 and TmHydF.^23^,^32^,^37^ This resulted in the incorporation of one [4Fe4S] cluster in each protein, with a total Fe content of 3.8 ± 0.3 (CrHydA1) and 3.4 ± 0.1 (CaHydF), and a total sulfide content of 3.4 ± 0.6 (CrHydA1) and 3.2 ± 0.2 (CaHydF), per monomer. In the as-prepared samples the [4Fe4S] clusters resided predominantly in their oxidized, EPR silent, state ([4Fe4S]_H_^2+^-CrHydA1 and [4Fe4S]^2+^-CaHydF), readily observable by UV/Vis spectroscopy (Fig. S3†). Still, EPR spectra recorded on the as-prepared proteins did also display axial signals attributable to their respective reduced [4Fe4S] clusters ([4Fe4S]_H_^+^-CrHydA1 and [4Fe4S]^+^-CaHydF),^11^,^26^ showing that a fraction of the clusters were reduced after reconstitution. Moreover, a relatively small axial EPR signal (*g* = 2.012) was discernible in the case of CaHydF prior to addition of NaDT. The latter signal disappeared upon reduction, indicating that a small portion of the reconstituted CaHydF protein contained a [3Fe4S] cluster rather than a [4Fe4S] cluster, most likely due to incomplete reconstitution. Conversely, the respective axial signals increased significantly in intensity upon NaDT treatment, in agreement with their assignment to [4Fe4S]_H_^+^-CrHydA1 and [4Fe4S]^+^-CaHydF (Fig. S3†).

### Assembly and characterization of [2Fe]^adt^-HydF

[2Fe]^adt^-CaHydF was prepared by treating the reconstituted CaHydF protein with a 12-fold molar excess of [2Fe]^adt^ at ambient temperature, followed by purification on a desalting column to remove unreacted [2Fe]^adt^. The yield of the reaction was approximately 70%, relative to CaHydF. As in the case of Tm and TmeHydF, the incorporation of the [2Fe]^adt^ complex into the protein was readily observable by the new absorption band at 350 nm, characteristic of the synthetic cofactor (Fig. S4†), as well an increase in total Fe content by 1.7 ± 0.3/CaHydF.^5^,^28^

EPR spectra recorded on as-prepared forms of [2Fe]^adt^-CaHydF did not reveal any new signal as compared to the spectrum observed for reconstituted CaHydF. Albeit, the intensity of both the [4Fe4S]^+^ as well as the [3Fe4S]^+^ signal (Fig. 2A) decreased significantly. Thus, EPR spectroscopy suggests that the [2Fe]^adt^ complex remains in its EPR silent Fe(i)Fe(i) state, in agreement with earlier studies of semi-synthetic HydF.^5^,^28^ Conversely, the incorporation of [2Fe]^adt^ into CaHydF resulted in distinct changes in the FTIR spectrum. The as-prepared sample displayed two bands in the cyanide region and four bands in the region associated with terminal CO ligands (Fig. 2B), clearly distinguishable from the spectrum of [2Fe]^adt^ in solution.^5^ Importantly, the observed spectrum is practically indistinguishable to the CaHydF^EG^ spectra reported by Shephard *et al.* and Scott *et al.*, apart from a minor shift of the weak feature at 1979 cm^–1^ in the latter case (Table 1).

**Table 1:** Vibrational frequencies (cm^–1^) reported for CaHydF

CN^–^	CO	Sample	Ref.
2047, 2024	1979, 1940, 1892, 1882	[2Fe]^adt^-CaHydF (as prepared)	This work
2068, 2047, 2025	1969, 1940, 1904, 1885	[2Fe]^adt^-CaHydF (reduced)^*a*^	This work
2068, 2044	1968, 1941, 1905, 1885	[2Fe]^adt^-CaHydF (oxidized)^*b*^	This work
2069, 2044	1967, 1943, 1907, 1877	CaHydF^EG^^*c*^	Czech *et al.*^25^
2047, 2027	1940, 1881	CaHydF^EG^^*d*^	Shepard *et al.*^26^
2050, 2026	1958, 1943, 1940, 1897, 1879	CaHydF^EG^^*d*^	Scott *et al.*^27^
2072, 2042, 2024	1971, 1948, 1943, 1909, 1888	CaHydF^EG^^*e*^ (reduced)	Scott *et al.*^27^

^*a*^[2Fe]^adt^-CaHydF, reduced by sodium-dithionite treatment.

^*b*^[2Fe]^adt^-CaHydF, oxidized by thionine acetate treatment.

^*c*^CaHydF^EG^ homologously expressed.

^*d*^CaHydF^EG^ heterologously expressed in *E. coli*.

^*e*^CaHydF^EG^ heterologously expressed in *E. coli*, reduced by sodium-dithionite treatment.

The reduction of [2Fe]^adt^-CaHydF resulted in the appearance of an axial, *S* = ½, EPR signal. The spectrum was highly similar to that observed for reconstituted CaHydF following NaDT reduction (Fig. 2C and S2†). The corresponding FTIR spectrum revealed only minor differences compared to the as-prepared sample (Fig. 2D). Still, it is noteworthy that the new features appearing at 1904 and 2068 cm^–1^ are in line with observations made for reduced forms of CaHydF^EG^.^27^ Additionally, the NaDT treatment resulted in a red-shift of the aforementioned weak feature at 1979 cm^–1^ to 1969 cm^–1^, and a corresponding feature is observed at 1971 cm^–1^ in reduced CaHydF^EG^.^27^ The EPR signal, in combination with the observed shifts in the FTIR spectrum, are attributed to a reduction of the [4Fe4S] cluster, while the [2Fe]^adt^ complex retained its(i,i) oxidation state ([Fe^I^Fe^I^]-[4Fe4S]^+^-CaHydF). The small changes in the FTIR spectrum upon reduction points towards a weak coupling between the [4Fe4S] cluster and [2Fe]^adt^ in [2Fe]^adt^-CaHydF, as compared to the H-cluster.

The possibility to oxidize the pre-catalyst on HydF was probed by incubating [2Fe]^adt^-CaHydF with thionine, a common oxidant for formation of the H_ox_-state in [FeFe] hydrogenase.^32^,^38^,^39^ This treatment resulted in a small shift of the CN bands to 2068 and 2044 cm^–1^ with a concomitant splitting of the low frequency CO bands to 1904 and 1885 cm^–1^ (Fig. 2F). Thereby generating a spectrum strikingly similar to that reported for homologously expressed CaHydF^EG^ by Czech *et al.* (Table 1). However, no new signal was observable in the EPR spectrum in the temperature range 10–20 K (Fig. 2E). Thus, there is no indication of oxidation of either the [4Fe4S] cluster or the HydF bound [2Fe]^adt^ complex. In contrast, treating an aqueous solution of [2Fe]^adt^ with thionine acetate resulted in a rapid loss of the absorbance at 350 nm, indicative of degradation of the complex (Fig. S5†). In summary, our combined EPR and FTIR characterization of [2Fe]^adt^-CaHydF underscores the biological relevance of this semi-synthetic protein, as we can reproduce the different reported FTIR spectra of CaHydF^EG^ within experimental error. By extension the data also lend further support to the hypothesis that the pre-catalyst on HydF is highly similar to [2Fe]^adt^.^5^,^28^ Moreover, our data shows that the cofactor is surprisingly resistant not only to reductants but also to chemical oxidants when bound to HydF.

### H-Cluster formation using [2Fe]^adt^-CaHydF

Our semi-synthetic HydF protein is also a functional mimic of HydF^EG^, as it is capable of activating apo-HydA1. The transfer of the pre-catalyst from [2Fe]^adt^-HydF to apo-HydA1 was studied by following the increase in hydrogenase activity upon mixing of the two proteins. As shown in Fig. 3A complete activation of CrHydA1 required the addition of a 10-fold excess of [2Fe]^adt^-CaHydF relative to apo-CrHydA1. Despite the requirement for an excess of [2Fe]^adt^-CaHydF, this form of HydF is still more efficient than isolated forms of biologically maturated CaHydF^EG^.^25^ Indeed, the observed stoichiometry is in good agreement with earlier studies using semi-synthetic forms of HydF from *T. maritima*.^5^ Thus, the biological origin of HydF does not appear to be a critical factor for the efficiency of interaction with apo-HydA.

In light of the structure of the H-cluster and the proposed structure of the pre-catalyst on HydF, the mechanism of H-cluster assembly is clearly a complex multi-step process. Key steps must include the release of the pre-catalyst from HydF, its transfer to the active site of HydA, the coordination of the cysteine derived thiol ligand and release of a CO ligand, resulting in the formation of the so-called “rotated structure”.^40^ The details of this reaction, as well as the order of events, remain to be elucidated. In order to gain further insight into this process we probed H-cluster assembly using a combination of CO-release assays and FTIR as well as EPR spectroscopy.

#### CO release during H-cluster assembly

The FTIR data reported by us and others, in combination with DFT calculations,^5^,^27^ indicate that the pre-catalyst is a 4 carbonyl species. Thus, the formation of an active HydA enzyme would require the release of a CO ligand. This reaction was monitored using a hemoglobin assay. Apo-CrHydA1 was incubated with various amounts of [2Fe]^adt^-CaHydF in gas tight vials for 30 min, after which time deoxyhemoglobin (Hb) was added to the reaction mixture. The CO release was thus determined *via* the formation of carboxyhemoglobin, COHb, detected using UV/Vis spectroscopy. The amount of CO released as a function of HydF equivalents was in good agreement with the enzymatic assay, and a plateau was reached after 10 equivalents of [2Fe]^adt^-CaHydF per apo-CrHydA1 (Fig. 3B).

The influence of time on the reaction was studied in a separate assay, in the time range of 5 to 45 minutes. As seen in Fig. 3C, even when mixing stoichiometric amounts of [2Fe]^adt^-CaHydF and apo-CrHydA1 (1 : 1 ratio), the release of CO appeared to happen on a minute time-scale. This observed time-dependence demonstrated that the inability to reach complete HydA activation using stoichiometric amounts of HydF was not attributable to a sluggish reaction, as the reaction had clearly reached completion after 30 minutes. Due to the nature of the assay, a detailed analysis of the initial phase of the reaction could not be performed. Conversely, it was possible to promote complete transfer of the pre-catalyst from HydF to apo-HydA1 by providing a large excess of apo-CrHydA1 (15 equivalents relative to [2Fe]^adt^-CaHydF, Fig. 3D).

#### Redox chemistry during H-cluster assembly

Electrochemical studies of the activation of apo-HydA using [2Fe]^adt^ in the absence of HydF have provided a model in which the assembly of the H-cluster results in the oxidation of the [2Fe] subsite precursor with concomitant reduction of the [4Fe4S]_H_ cluster to form an initial [4Fe4S]_H_^+^–[Fe^I^Fe^II^] species (denoted H_red_′).^41^ In order to probe possible redox state changes during [2Fe]^adt^-CaHydF mediated activation, we studied the reaction by FTIR and EPR spectroscopy. In order to ensure efficient transfer of the pre-catalyst from HydF, an excess of apo-HydA1 was used. The protein mixtures were incubated under strict anaerobic conditions for 60 min before samples were either immediately analyzed by FTIR or transferred to EPR tubes and flash frozen. The resulting FTIR spectra revealed the presence of predominantly two species, assigned to the H_ox_ state (1963 and 1939 cm^–1^) and the corresponding CO-inhibited, H_ox_-CO, form (2013, 1968, 1963 and 1808 cm^–1^) of the H-cluster (Fig. 4B).^42^,^43^ No signals related to reduced states of the H-cluster were discernible, nor any residual signal associated with [2Fe]^adt^-CaHydF. In the case of H_ox_, the H-cluster resides in a [4Fe4S]_H_^2+^-[Fe^I^Fe^II^] state. Thus, the formation of this species requires a one-electron oxidation of the pre-catalyst. The oxidation was not attributable to proton reduction, as no hydrogen gas was detected in the headspace gas when the reaction was performed in sealed vials.

In accordance with the FTIR experiments, a mixture of signals arising from H_ox_ (reported *g* values = 2.10, 2.037 and 1.996) and H_ox_-CO (reported *g* values = 2.052 and 2.007) was observed in the EPR spectrum (Fig. 4C).^42^,^44^ Moreover, a significant increase of the trough at *g* = 1.87 was detected (Fig. 4B inset). The *g* = 1.87 signal is attributable to [4Fe4S]^+^-CaHydF, showing that a reduction of CaHydF occurred during the reaction. More specifically, the EPR spectra were recorded on samples in which 200 μM apo-CrHydA1 had been incubated with 30 μM [2Fe]^adt^-CaHydF, and quantification of the *g* = 1.87 signal using a dithionite reduced reference sample of CaHydF (30 μM, [4Fe4S]^+^-CaHydF) shows that the signal represents 16 ± 2 μM [4Fe4S]^+^-CaHydF (Fig. 4 and Fig. S6†). As no external reductant was added to the reaction, we attribute this reduction to an electron transfer from the H-cluster upon formation of the H_ox_ state. EPR samples collected at shorter time intervals revealed a mixture of the H_ox_ and H_ox_-CO states after 6 minutes. The latter species decreased in intensity over 10 minutes to yield a spectrum enriched in H_ox_ (Fig. S7†). The final predominance of H_ox_-CO observed in the 60 minutes spectrum is attributed to reformation of the CO inhibited state upon extended incubation times.^45^ In summary, our FTIR and EPR data support the hypothesis that an oxidation of the [2Fe] subsite occurs during H-cluster assembly. Additionally, the observed reduction of CaHydF suggests that this protein serves as an electron acceptor in the process.

**Scheme 1:**
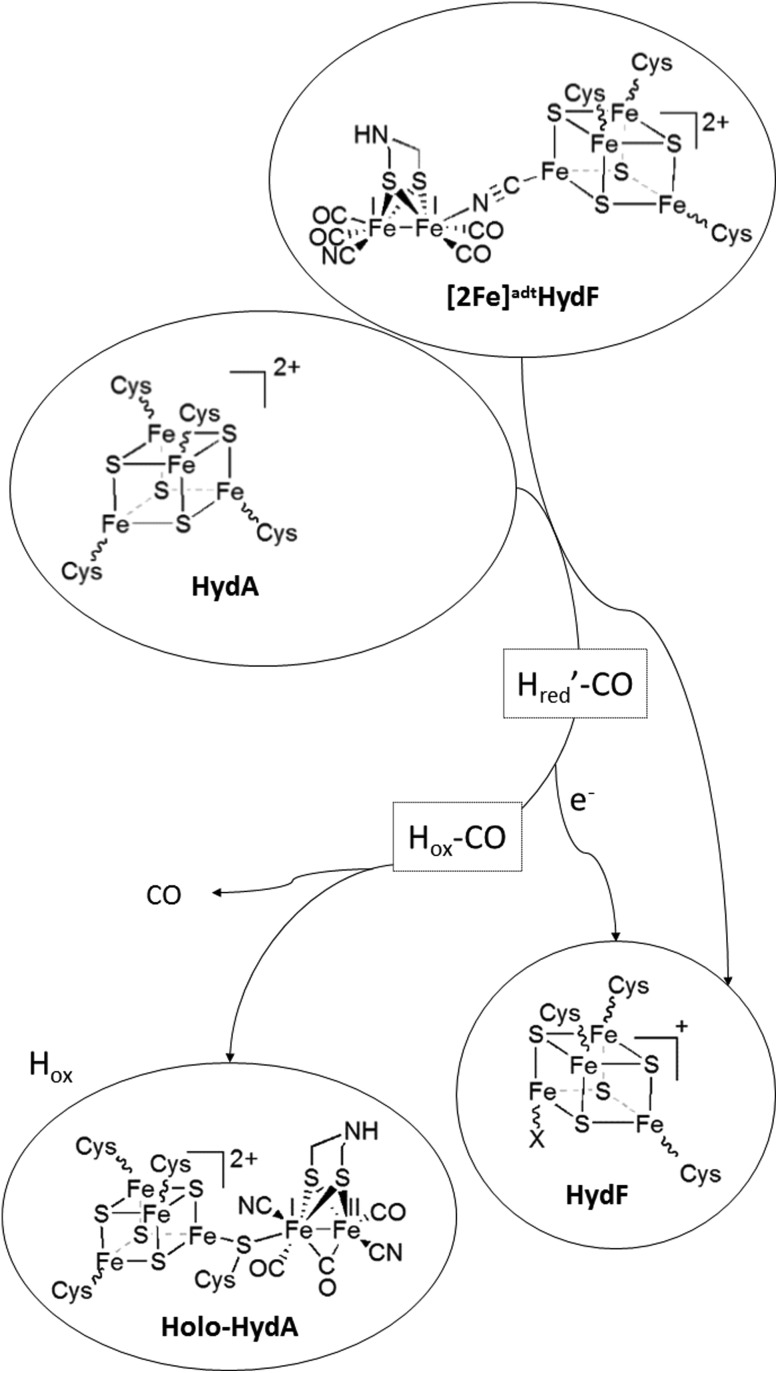
Schematic representation of H-cluster assembly. The transfer of the pre-catalyst ([2Fe]^adt^) from HydF results in the release of a CO ligand and oxidation of the dinuclear complex to give H_ox_ ([Fe^II^Fe^I^]-[4Fe4S]^2+^-HydA), with concomitant reduction of the [4Fe4S] cluster on HydF ([4Fe4S]^+^-HydF). Note that the model for [2Fe]^adt^ binding to HydF is based on ref. 5. An alternative model, with a reversed μ-CN^–^ bond is proposed in ref. 27.

The aforementioned electrochemical study on H-cluster assembly proposed that the initial state formed is similar, or identical, to H_red_′.^41^ On a longer time-scale we observe H_ox_ and H_ox_-CO as the dominant states by FTIR spectroscopy, when we perform the reaction in the absence of external reductants or hemoglobin. Moreover, our EPR data suggest that the formation of H_ox_ is delayed relative to H_ox_-CO. A plausible mechanism for H-cluster assembly would then proceed *via* initial formation of a CO inhibited H_red_-like species (H_red_′–CO), recently observed *via* reduction of H_ox_-CO.^46^ We have not yet been able to trap this reduced intermediate, arguably due to rapid oxidation of the H-cluster to give H_ox_-CO, with concomitant reduction of HydF. This electron transfer event is then followed by the release of the fourth CO ligand present on the [2Fe] pre-catalyst. The resulting model for H-cluster assembly is summarized in Scheme 1.

## Conclusions

In summary, our results underscore the biological relevance of artificially matured HydF. Moreover, we employ this semi-synthetic form of HydF to verify that the pre-catalyst is truly a four CO species, as indicated by FTIR spectroscopy and that the pre-catalyst is remarkably stable towards both potent oxidants and reductants when bound to HydF, suggesting a protective role of HydF during the assembly process. Finally, based on our combined FTIR and EPR study of the H-cluster assembly reaction we propose that the [4Fe4S] cluster present in HydF is not only responsible for the binding of the pre-catalyst to HydF, but is also directly involved in H-cluster assembly by acting as an electron acceptor in the reaction. This is expected to facilitate H_ox_ formation and thus ensuring the irreversibility of the transfer reaction.

## Conflicts of interest

There are no conflicts to declare.

## Supplementary Material

Supplementary information
