# Supplementary material for: Monitoring H-cluster assembly using a semi-synthetic HydF protein
Source: Dalton Trans. 2019 Jan 3;48(18):5978–86. doi: 10.1039/c8dt04294b (PMC6509880; doi:10.1039/c8dt04294b)
Supplement: Supplementary file 1 [file DT-048-C8DT04294B-s001.pdf]

Electronic Supplementary Material (ESI) for Dalton Transactions.
This journal is © The Royal Society of Chemistry 2019

Monitoring H-cluster assembly using semi-synthetic HydF

Brigitta Németh, Charlène Esmieu, Holly J. Redman and Gustav Berggren

Supporting information

Table of Contents

Figure S1. The effect of the baseline treatment on the FTIR spectra.
Figure S2. Hemoglobin calibration curves.
Figure S3. UV/Vis and EPR spectra recorded on CrHydA1 and CaHydF after reconstitution.
Figure S4. Monitoring $[2Fe]^{adt}$-HydF formation by UV/Vis spectroscopy.
Figure S5. Monitoring the reaction between $[2Fe]^{adt}$ and thionine by UV/Visible spectroscopy.
Figure S6. Quantification of HydF reduction during H-cluster assembly using EPR spectroscopy.
Figure S7. Monitoring H-cluster assembly over time by EPR spectroscopy.

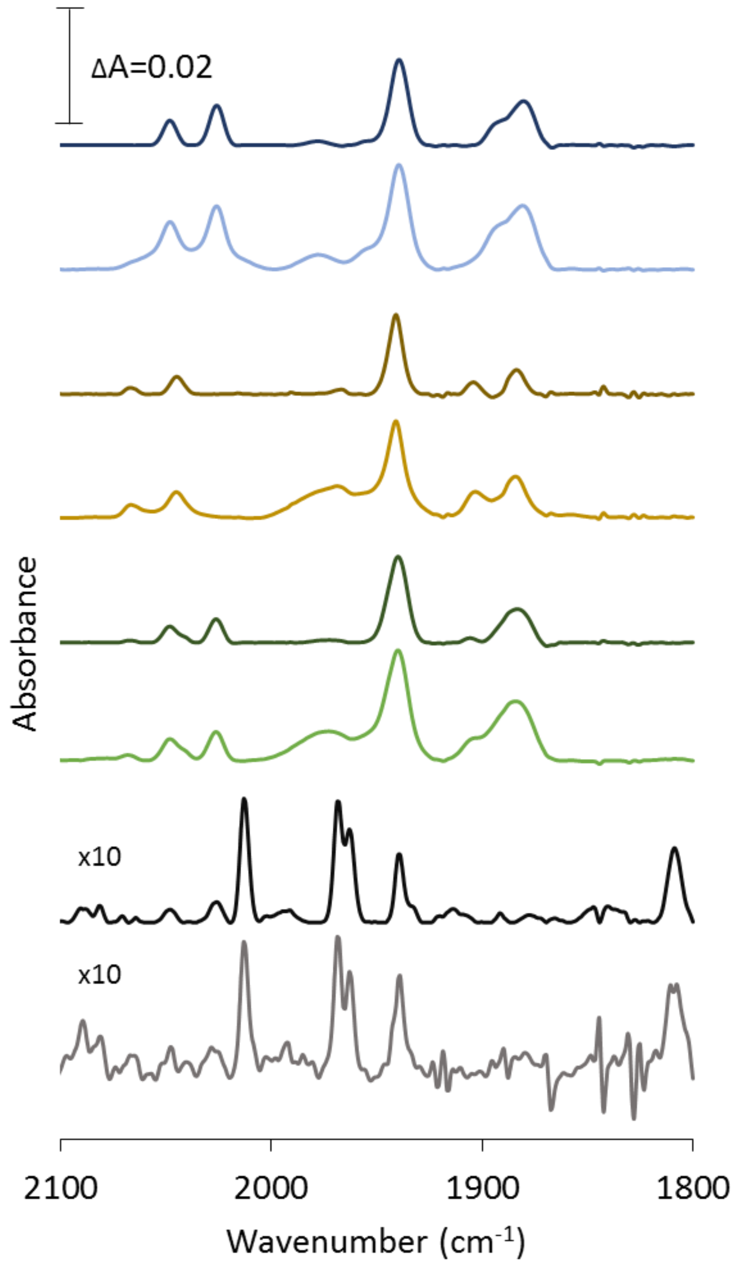

Figure S1. The effect of the baseline treatment on the FTIR spectra.
The rubber-band concave (RBC) baseline treatment was carried out in OPUS (Bruker), the manual polynomial (MP) baseline treatment was carried out in Origin. From top to bottom: $[2Fe]^{adt}$-CaHydF as prepared RBC (dark blue), $[2Fe]^{adt}$-CaHydF as prepared MP (light blue); $[2Fe]^{adt}$-CaHydF thionine acetate oxidized RBC (dark yellow), $[2Fe]^{adt}$-CaHydF thionine acetate oxidized MP (light yellow); $[2Fe]^{adt}$-CaHydF Na-DT reduced RBC (dark green), $[2Fe]^{adt}$-CaHydF Na-DT reduced MP (light green); $[2Fe]^{adt}$-CaHydF + apo-CrHydA1 RBC (black), $[2Fe]^{adt}$-CaHydF + apo-CrHydA1 as prepared MP (black). The two baseline treatment protocols result in limited changes to relative peak intensities and the peak shape, while peak position remain constant. Sample conditions are given in Figure 2 and 4 of the main text. Due to low sample concentration and signal intensity, the two latter spectra were multiplied by the factor of 10.

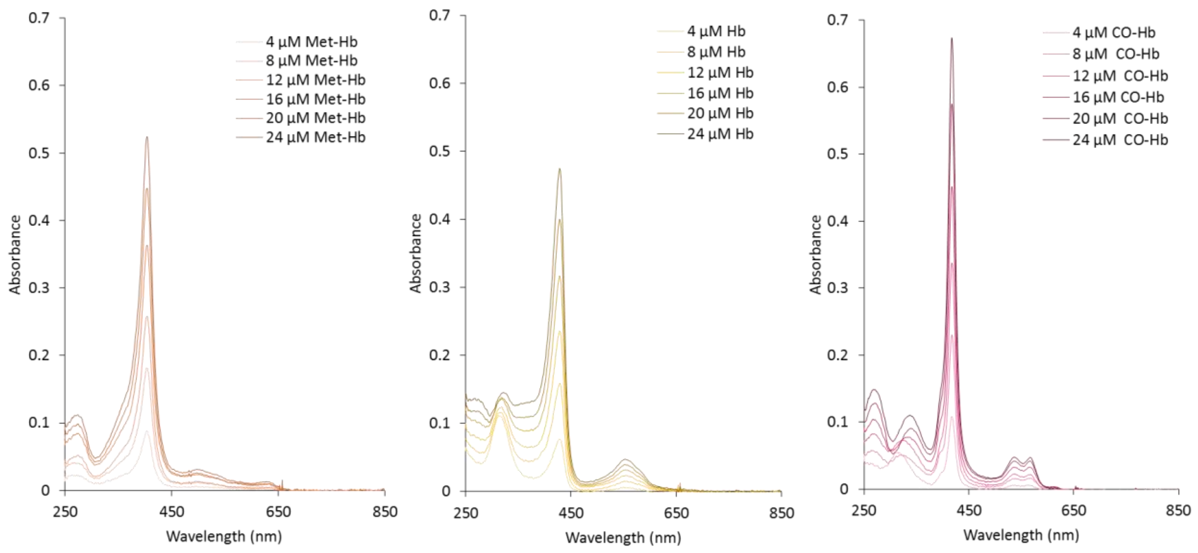

Figure S2. Hemoglobin calibration curves. Methemoglobin, deoxyhemoglobin (Hb) and carboxyhemoglobin (CO-Hb) calibration curves. 100 µM met-hemoglobin solution was prepared from bovine hemoglobin (Sigma) dissolved in 100 mM K-phosphate buffer pH 6.8. Dilution curve was prepared in the range of 1-6 µM hemoglobin concentrations in 100 mM K-phosphate buffer pH 6.8. Each methemoglobin sample was measured by UV/Vis (hemoglobin ox). Deoxyhemoglobin was generated via the addition of 100 µM (final concentration) of Na-DT to each methemoglobin sample. After a 5 minute incubation period the UV/Vis spectrum was recorded for each sample (hemoglobin red). To obtain the carboxyhemoglobin form, the deoxyhemoglobin samples were transferred into gas tight serum vials and flushed with pure CO gas for 5 minutes. The vials were opened in the glovebox and their respective UV/Vis spectra were recorded (hemoglobin CO). The spectra were recorded with a l=0.2 cm cuvette.

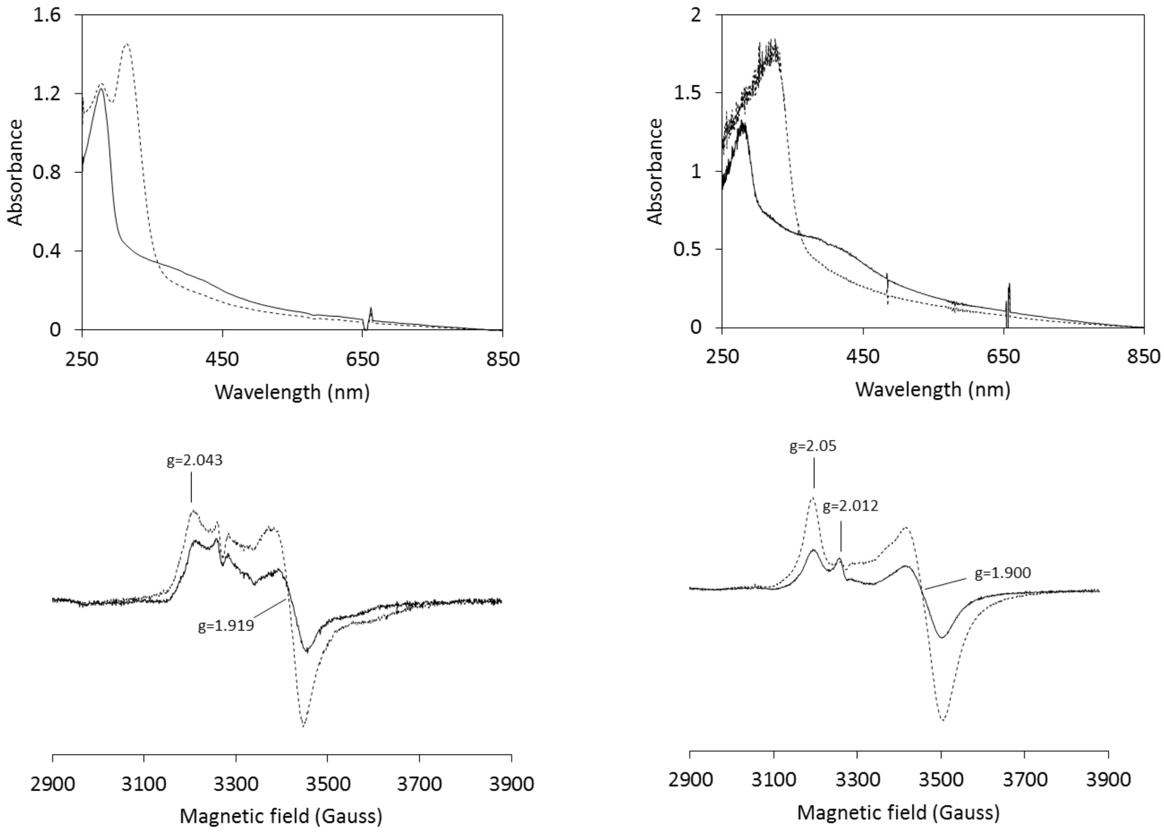

Figure S3. UV/Vis and EPR spectra recorded on CrHydA1 and CaHydF after reconstitution. Left panel: (top) UV/Vis spectra of CrHydA1 (100 µM) after reconstitution in the absence (black solid line) or presence of dithionite (2 mM, black dashed line); (bottom) EPR spectra of CrHydA1 (200 µM) in the absence (black solid line) or presence of dithionite (4 mM, black dashed line). Right panel: (top) UV/Vis spectra of CaHydF (200 µM) after reconstitution in the absence (black solid line) or presence of dithionite (2 mM, black dashed line); (bottom) EPR spectra of CaHydF (200 µM) in the absence (black solid line) or presence of dithionite (2 mM, black dashed line). EPR spectra recorded at 10K, microwave power 1 mW. Microwave frequency: 9.28 GHz. All samples were prepared in 100 mM Tris-HCl 300 mM KCl 25 mM $MgCl_2$ buffer. The spectra were recorded with a l=0.2 cm cuvette.

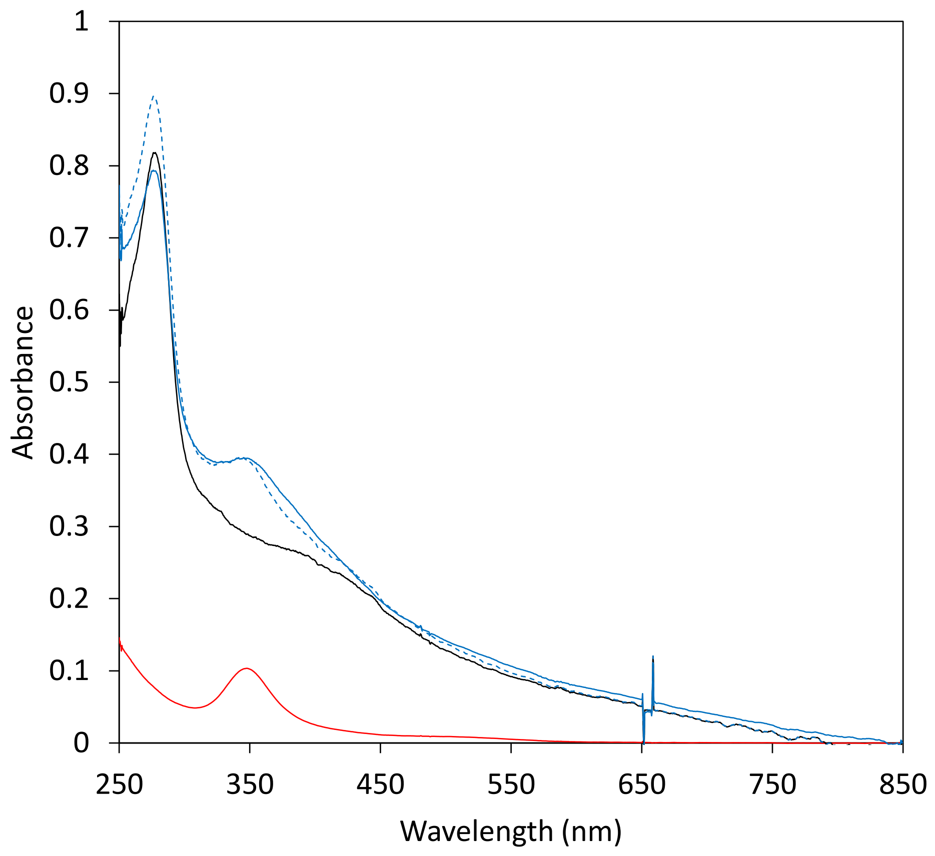

Figure S4. Monitoring [2Fe]^{adt}-HydF formation by UV/Vis spectroscopy. The solid lines represent experimental data, while the dashed line represent a linear combination of two experimental spectra. 130 µM [4Fe4S]$^{2+}$-CaHydF (solid black), 130 µM [2Fe]^{adt}-CaHydF (solid blue line); 130 µM [2Fe]^{adt} (solid red); A linear combination of 130 µM [2Fe]^{adt} and 130 µM [4Fe4S]$^{2+}$-CaHydF spectrum (dashed blue). All spectra were recorded in 100 mM Tris-HCl pH 8.0, 300 mM KCl. The spectra were recorded with a l=0.2 cm cuvette.

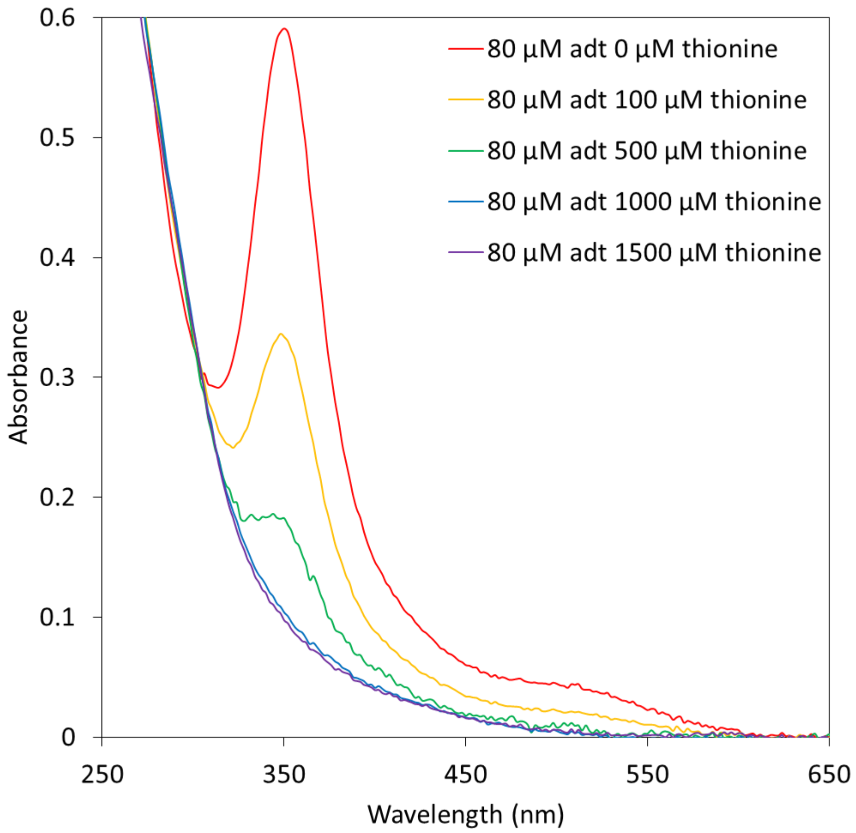

Figure S5. Monitoring the reaction between $[2Fe]^{adt}$ and thionine by UV/Visible spectroscopy. 80 µM $[2Fe]^{adt}$ complex was treated with various amounts (100-1500 µM, 1.25-18.75 equivalents) of thionine acetate for 5 minutes under strict anaerobic conditions in the glovebox at room temperature under dimmed light conditions, resulting the disappearance of the characteristic absorption band at 350 nm. The $[2Fe]^{adt}$ complex was dissolved in 100 mM Tris-HCl pH 8.0, 300 mM KCl, 25 mM $MgCl_2$. The solid thionine acetate powder was dissolved in 100 mM Tris-HCl pH 8.0, 300 mM KCl, 25 mM $MgCl_2$. The excess solid thionine acetate particles were removed via filtration before recording the UV/Vis spectra. The spectra were recorded with a l=1 cm cuvette.

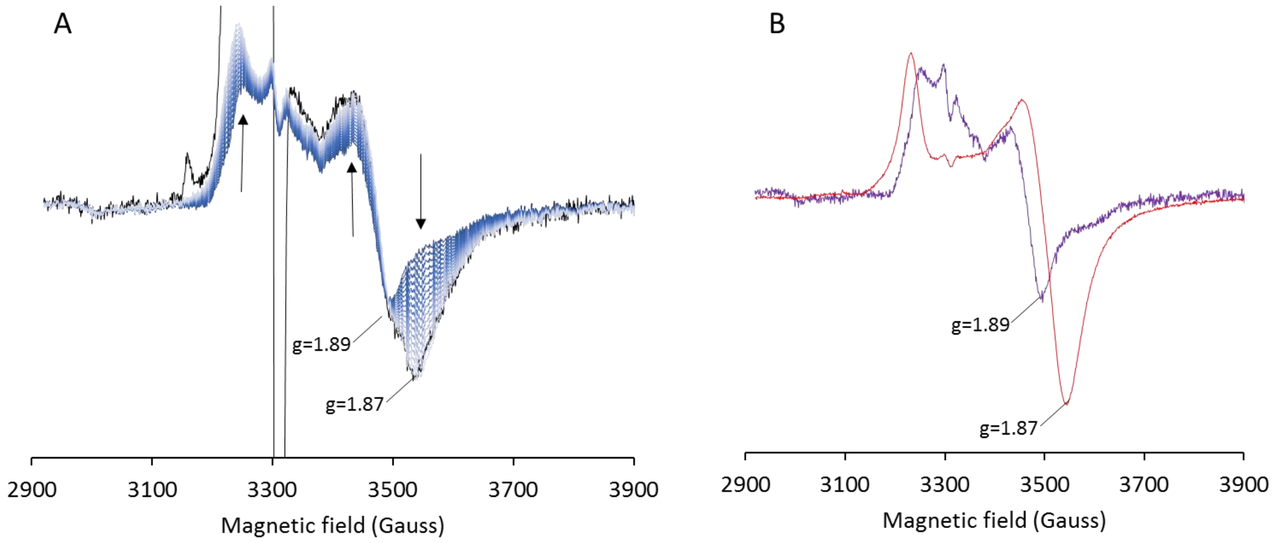

Figure S6. Quantification of HydF reduction during H-cluster assembly using EPR spectroscopy. Panel A: Low temperature X-band EPR spectrum recorded on a mixture of 200 µM CrHydA1 and 30 µM $[2Fe]^{adt}$-CaHydF incubated for 60 minutes, recorded at 10 K with 1 mW microwave power, microwave frequency: 9.28 GHz. (black solid line); and a reproduction of the experimental spectrum through a linear combination of CrHydA1 (as-prepared, 200 µM) and increasing concentrations (1µM-16 µM, shown as dark to pale blue) of reduced CaHydF ($[4Fe4S]^{+}$-CaHydF). The H_{ox} and H_{ox}-CO signals observable at $g \geq 2$ are not included in the fitting.

Panel B: Representative spectra of $[4Fe4S]^{+}$-CrHydA1 (as-prepared, 200 µM) (purple solid line) and 30 µM dithionite treated CaHydF ($[4Fe4S]^{+}$-CaHydF, red solid line) used in the fitting procedure shown in panel A. Troughs at g = 1.89 ($[4Fe4S]^{+}$-CrHydA1) and 1.87 ($[4Fe4S]^{+}$-CaHydF) indicated for clarity.

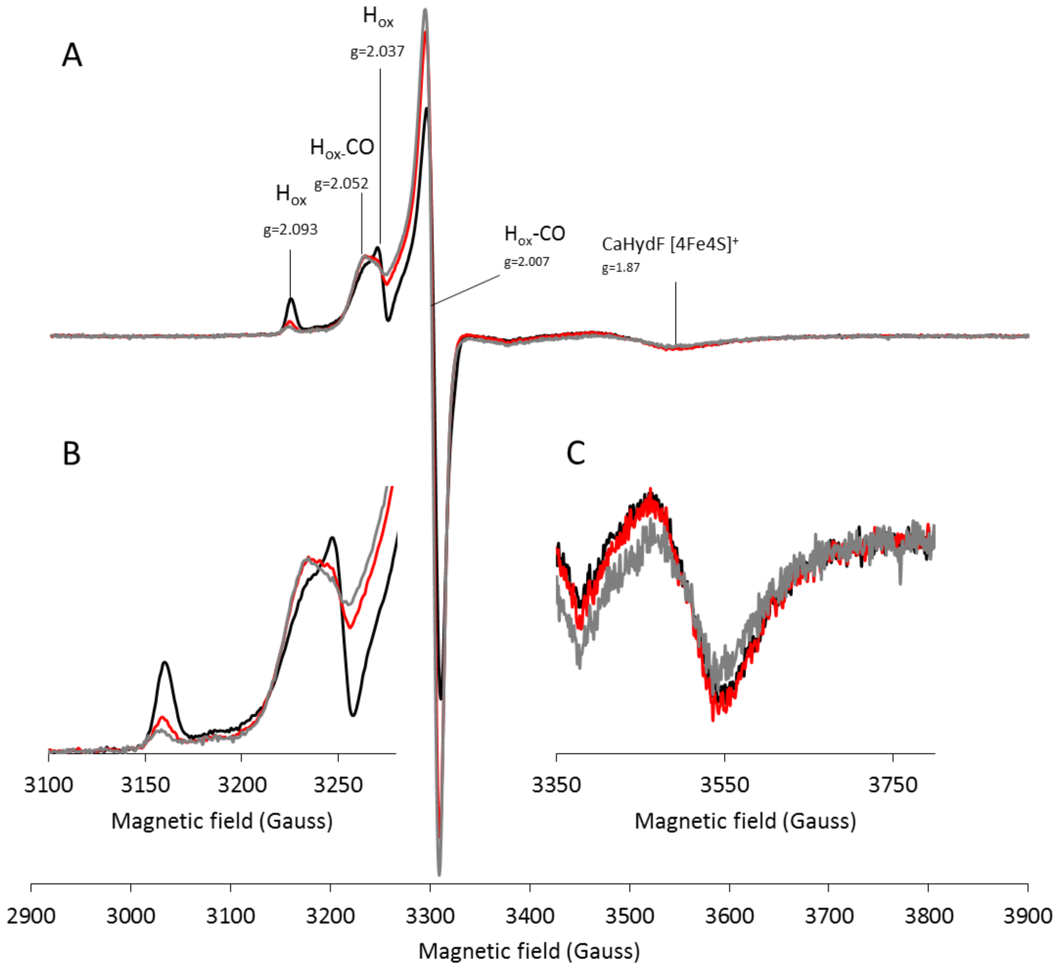

Figure S7. Monitoring H-cluster assembly over time by EPR spectroscopy. A mixture of apo-CrHydA1 (200 µM) and $[2Fe]^{adt}$-CaHydF (30 µM) was prepared under anaerobic conditions, and EPR samples collected after 6 (black spectrum), 16 (red spectrum) and 60 (grey spectrum) minutes were flash frozen in liquid nitrogen. There is an increase in the H_{ox} signature observable between the 6 and 16 minute spectra, with a concomitant decrease of the H_{ox}-CO features. Extended incubation times (60 minutes spectrum) transforms the majority of the sample back into H_{ox}-CO. Panel A: Full scan window, highlighting changes in the intense H_{ox}-CO feature at g= 2.007; Panel B: Zoom in of low field region (3100-3300 Gauss), highlighting changes in the characteristic peak of H_{ox} at g = 2.10; Panel C: Zoom in of high field region (3350-3650 Gauss), highlighting changes in the g = 1.87 signal attributed to $[4Fe4S]^{+}$-CaHydF. HydF becomes reduced within minutes, and only minor changes are observed upon extended incubation.

Spectra were recorded at recorded at 10 K with 1 mW microwave power, microwave frequency: 9.28 GHz.
